# Supplementary material for: Does financial asset allocation term structure affect audit fees? Evidence from China
Source: PLoS One. 2025 Jan 17;20(1):e0317671. doi: 10.1371/journal.pone.0317671 (PMC11741647; doi:10.1371/journal.pone.0317671)
Supplement: Attachment — Submitted filename: Response to Reviewers.docx [file pone.0317671.s002.docx]

**Response to Reviewers**

**Dear Editors and Reviewers:**

Thanks a lot for your letter and the reviewers’ comments concerning our manuscript entitled “*Financial asset allocation term structure, Independent directors Characteristics and Audit fees: Evidence from China*”. We are happy to respond to your comments and suggestions, and grateful to take this as a great opportunity to update our information and try our best to meet the high standards of ***PLOS ONE***. The main corrections in the paper and the responses to the editor’s and reviewers’ comments, point-by-point, are as follows:

**Editor comments:**

1.After my own reading of the study, I agree with the reviewer's evaluation. I would like to highlight that the theoretical foundation regarding audit fees requires further development in its current form. There exists a substantial body of literature on the determinants of audit fees that has been well-established over several decades, and I would encourage its incorporation in your revision. In particular, the link between asset risk and audit fees would benefit from a deeper analysis.

**R: Thank you so much!**

We believe that your valuable comments are very helpful in improving the quality of the manuscript and have expanded our research vision and research ideas, giving us a deeper knowledge of current research. Based on your comments, we have strengthened the analysis of the link between asset risk and audit fees in the introduction and theoretical research sections. References to more compelling articles on current research have been updated in the body of the manuscript and references.

Firstly, we updated our analysis of the link between asset risk and audit fees in the introduction:

Real enterprises are facing the dilemma of shrinking market, intensifying competition, overcapacity, profit compression, and declining return on investment [1, 2]. In the background of risk-oriented auditing, scholars have gradually begun to explore the relationship between financial asset allocation and audit fees. Audit fees theory holds that audit fee mainly depends on two parts: cost input and risk premium in the audit process [3]. If the auditor enhances the assessment of the risk of the enterprise, it will lead to an increase in the content and complexity of the audit work and raise the audit fee [4, 5]. Distinguished from the general main business, there is a serious agency conflict hidden in the process of financial asset allocation by enterprises [6], which leads to the financial asset allocation becoming a tool for management to glorify the performance and earnings management, and exacerbates the operational and financial risks of the enterprise [7], so in the audit of the financial asset allocation of the enterprise, it requires experienced auditors to carry out the audit. At the same time, it increases the complexity of corporate auditing. Especially for the assessment of enterprise asset risk, it will affect the audit cost of the enterprise [8].

The current study of financial asset allocation motives mainly includes the saving motive to prevent risk, and the profit-seeking motive to obtain more returns [9, 10]. Financial assets can be divided into short-term and long-term according to their term structure, which makes the financialization of enterprises show different characteristics of the term structure [11]. According to the theory of capital demand, short-term financial assets, as a substitute for cash, have strong liquidity and liquidity ability, and the trading financial assets held by enterprises are a buffer to deal with financial crisis, which can prevent corporate risk [12], and are not the main factor to enhance corporate audit fees. Long-term financial assets are mostly long-term investments with high returns, in line with the profit-seeking motive of financial asset allocation [11]. High returns imply high risks, and long-term financial asset allocation will expose enterprises to more risks due to the long cycle, more complex business structure, and high degree of uncertainty in the internal and external environments they face [4, 8]. When the enterprise faces the need to replenish the funds required by the unexpected situation in the short term, the long-term financial assets lack sufficient liquidity, and it is difficult for the enterprise to change the nature of the funds in a timely manner in the face of the financing constraints and investment opportunities, and at the same time, too many long-term financial asset allocations increase the complexity of the auditing business, which in turn exacerbates the level of the risk of the assets faced by the entity enterprise, thus enhancing the audit costs [8].

Secondly, we have added to the introduction a section about the relationship between financial asset allocation and financial risk:

Financial risk is an important factor affecting the audit fee of enterprises[8]. In order to make an objective and fair audit report, the auditor will carry out a lot of audit work to find out the enterprises' financial risks [7]. At the same time, the managers of the enterprise will cover up the financial risks of the enterprise, which increases the difficulty of the audit work. When the enterprise faces higher financial risks, it will increase the audit fees[18]. The main purpose of the enterprise's financial asset allocation is to increase earnings, which will bring financial risk. In the term structure of financial asset allocation, long-term financial assets are characterized by high return and high risk [11], indicating that this effect mainly comes from long-term financial asset allocation, so financial risk will have an impact between the term structure of financial assets and audit costs.

At the same time, We have emphasized in the theoretical analysis and research hypotheses section that asset risk from long-term financial asset allocations is a significant cause of higher audit fees:

There are two main motives for corporate financial asset allocation: the preventive saving motive and the profit-chasing motive [19]. Preventive saving motive is mainly manifested in alleviating financing constraints by allocating more liquid short-term financial assets to cope with the enterprise's capital demand in unexpected situations, which will reduce the enterprise's asset risk; profit-chasing motive is manifested in allocating less liquid but higher-returning financial assets to obtain the excess rate of return [20], which will enhance the enterprise's asset risk. Based on the risk orientation of audit fees [18], the main reason for increasing audit fees should be the level of long-term financial asset allocation in financial asset allocation.

Finally, we refer to recent studies in recent years.

Thanks again and Best regards!

**Reviewers' comments:**

Reviewer #1:

1. The authors should enhance the motivation of the study, it is why to study association of financial assets on audit costs, and why utilize the Chinese context to analyze this issue. The paper does not have strong research significance and these issues need to be highlighted in the research contribution.

**R: Thank you so much!**

Your comments are very important to the quality of our article, and we are honored to have been able to improve it with your help. We have strengthened the motivation of the study in the introduction and theoretical analysis. Meanwhile, the significance of the study was elaborated in the research contribution, and changes were made in the manuscript as follows:

Firstly, we enhance the motivation of the study in Introduction section:

Chinese real enterprises are facing the dilemma of shrinking market, intensifying competition, overcapacity, profit compression, and declining return on investment [1, 2]. In the background of risk-oriented auditing, scholars have gradually begun to explore the relationship between financial asset allocation and audit fees. Audit fees theory holds that audit fee mainly depends on two parts: cost input and risk premium in the audit process [3]. If the auditor enhances the assessment of the risk of the enterprise, it will lead to an increase in the content and complexity of the audit work and raise the audit fee [4, 5]. Distinguished from the general main business, there is a serious agency conflict hidden in the process of financial asset allocation by enterprises [6], which leads to the financial asset allocation becoming a tool for management to glorify the performance and earnings management, and exacerbates the operational and financial risks of the enterprise [7], so in the audit of the financial asset allocation of the enterprise, it requires experienced auditors to carry out the audit. At the same time, it increases the complexity of corporate auditing. Especially for the assessment of enterprise asset risk, it will affect the audit cost of the enterprise [8].

The current study of financial asset allocation motives mainly includes the saving motive to prevent risk, and the profit-seeking motive to obtain more returns [9, 10]. Financial assets can be divided into short-term and long-term according to their term structure, which makes the financialization of enterprises show different characteristics of the term structure [11]. According to the theory of capital demand, short-term financial assets, as a substitute for cash, have strong liquidity and liquidity ability, and the trading financial assets held by enterprises are a buffer to deal with financial crisis, which can prevent corporate risk [12], and are not the main factor to enhance corporate audit fees. Long-term financial assets are mostly long-term investments with high returns, in line with the profit-seeking motive of financial asset allocation [11]. High returns imply high risks, and long-term financial asset allocation will expose enterprises to more risks due to the long cycle, more complex business structure, and high degree of uncertainty in the internal and external environments they face [4, 8]. When the enterprise faces the need to replenish the funds required by the unexpected situation in the short term, the long-term financial assets lack sufficient liquidity, and it is difficult for the enterprise to change the nature of the funds in a timely manner in the face of the financing constraints and investment opportunities, and at the same time, too many long-term financial asset allocations increase the complexity of the auditing business, which in turn exacerbates the level of the risk of the assets faced by the entity enterprise, thus enhancing the audit costs [8]. Therefore, the main reason why financial asset allocation affects the audit fee is the risk posed. This study tries to investigate whether it is the short-term or long-term financial asset allocation that brings risks to enterprises from the perspective of the term structure of financial asset allocation and analyze the inner mechanism.

Secondly，we explain why utilize the Chinese context to analyze this issue in introduction section.

Chinese real enterprises are facing the dilemma of shrinking market, intensifying competition, overcapacity, profit compression, and declining return on investment [1, 2].

Independent directors Characteristics refer to the distribution of independent directors' power shaped by institutional regulations or organizational design, defining the multidimensional situation of the board's structure and status, which are designed to ensure that the independent directors provide the board with a more professional and independent decision-making and oversight capacity within the framework of the formal power, and play a decisive role in strategy formulation and resolving procedural uncertainties [13-15]. Based on modern organizational theory, the effectiveness of board governance affects the structural configuration of corporate assets. Good independent directors governance can improve the efficiency of corporate decision-making [16], enhance the effectiveness of asset allocation decisions, and optimize corporate asset structure allocation. In recent years, there have been frequent cases of financial fraud by listed companies in China. The government and investors have increasingly emphasized the monitoring and decision-making role of independent directors. Thus, independent directors governance may have an impact on the relationship between financial asset allocation term structure and audit fees. However, the direction and extent of this influence are constrained by the independent directors' own characteristics. As a result, different independent directors' characteristics may have different impacts on the relationship between financial asset allocation term structure and corporate audit fees[17].

Thirdly, we highlighted research significance in the research contribution.

The possible contributions, firstly, this study explores the relationship between financial asset allocation and audit fees from two dimensions: short-term financial assets and long-term financial assets, expanding the research on the relationship between financial asset allocation and audit fees. Secondly, based on the risk orientation of audit fees, this study finds that in the term structure of financial asset allocation, long-term financial asset allocation leads to the fact that firms will be exposed to higher financial risks, which is the main reason for the increase in firms' audit fees, and enriches the literature on the differences in the level of audit fees under the risk-oriented perspective. Thirdly, it expands the related research on independent director characteristics and explores the role of independent director characteristics in decision-making and monitoring when firms engage in financial asset allocation, which provides empirical experience for preventing the risk of firms' financial asset allocation.

2. The research findings should be clearly written in the Introduction.

**R: Thank you so much!**

The research findings have been described in the introduction section and changes have been made in the manuscript as follows:

Based on the above analysis, using a sample of Chinese A-share listed companies in the period 2009-2019 as the research sample, this study examines the relationship between the term structure of financial asset allocation and audit fees, as well as the moderating role of independent directors' characteristics and the mediating role of financial risk. It is found that the relationship between short-term financial asset allocation and audit fees is insignificant, and long-term financial asset allocation significantly contributes to audit fees and is the main reason for the increase in corporate audit fees, and this effect is more significant in large firms and firms with high financing constraints. The proportion of female independent directors and financial background negatively moderated the relationship between long-term financial assets and audit fees, and overseas background and academic background positively moderated the positive relationship between long-term financial assets and audit fees. Financial risk plays a partial mediating role.

3. The analysis of auditing in theoretical analysis and research hypotheses is insufficient, and the reasons for the differences in the level of audit fees should be analyzed from the perspective of risk-oriented auditing.

**R: Thank you so much!**

Based on your comments, we have analyzed the reasons for the level of audit fee variance from a risk-oriented perspective and have expressed them in the Introduction section and Theoretical Analysis and Research Hypotheses section as follows:

Firstly, we updated our analysis of the link between asset risk and audit fees in the introduction:

Chinese real enterprises are facing the dilemma of shrinking market, intensifying competition, overcapacity, profit compression, and declining return on investment [1, 2]. In the background of risk-oriented auditing, scholars have gradually begun to explore the relationship between financial asset allocation and audit fees. Audit fees theory holds that audit fee mainly depends on two parts: cost input and risk premium in the audit process [3]. If the auditor enhances the assessment of the risk of the enterprise, it will lead to an increase in the content and complexity of the audit work and raise the audit fee [4, 5]. Distinguished from the general main business, there is a serious agency conflict hidden in the process of financial asset allocation by enterprises [6], which leads to the financial asset allocation becoming a tool for management to glorify the performance and earnings management, and exacerbates the operational and financial risks of the enterprise [7], so in the audit of the financial asset allocation of the enterprise, it requires experienced auditors to carry out the audit. At the same time, it increases the complexity of corporate auditing. Especially for the assessment of enterprise asset risk, it will affect the audit cost of the enterprise [8].

The current study of financial asset allocation motives mainly includes the saving motive to prevent risk, and the profit-seeking motive to obtain more returns [9, 10]. Financial assets can be divided into short-term and long-term according to their term structure, which makes the financialization of enterprises show different characteristics of the term structure [11]. According to the theory of capital demand, short-term financial assets, as a substitute for cash, have strong liquidity and liquidity ability, and the trading financial assets held by enterprises are a buffer to deal with financial crisis, which can prevent corporate risk [12], and are not the main factor to enhance corporate audit fees. Long-term financial assets are mostly long-term investments with high returns, in line with the profit-seeking motive of financial asset allocation [11]. High returns imply high risks, and long-term financial asset allocation will expose enterprises to more risks due to the long cycle, more complex business structure, and high degree of uncertainty in the internal and external environments they face [4, 8]. When the enterprise faces the need to replenish the funds required by the unexpected situation in the short term, the long-term financial assets lack sufficient liquidity, and it is difficult for the enterprise to change the nature of the funds in a timely manner in the face of the financing constraints and investment opportunities, and at the same time, too many long-term financial asset allocations increase the complexity of the auditing business, which in turn exacerbates the level of the risk of the assets faced by the entity enterprise, thus enhancing the audit costs [8].

Secondly, we have added to the introduction a section about the relationship between financial risk and audit fee:

Financial risk is an important factor affecting the audit fee of enterprises[8]. In order to make an objective and fair audit report, the auditor will carry out a lot of audit work to find out the enterprises' financial risks [7]. At the same time, the managers of the enterprise will cover up the financial risks of the enterprise, which increases the difficulty of the audit work. When the enterprise faces higher financial risks, it will increase the audit fees[18]. The main purpose of the enterprise's financial asset allocation is to increase earnings, which will bring financial risk. In the term structure of financial asset allocation, long-term financial assets are characterized by high return and high risk [11], indicating that this effect mainly comes from long-term financial asset allocation, so financial risk will have an impact between the term structure of financial assets and audit costs.

At the same time, We have emphasized in the theoretical analysis and research hypotheses section that risk-oriented from financial asset allocations is a significant cause of audit fees:

There are two main motives for corporate financial asset allocation: the preventive saving motive and the profit-chasing motive [19]. Preventive saving motive is mainly manifested in alleviating financing constraints by allocating more liquid short-term financial assets to cope with the enterprise's capital demand in unexpected situations, which will reduce the enterprise's asset risk; profit-chasing motive is manifested in allocating less liquid but higher-returning financial assets to obtain the excess rate of return [20], which will enhance the enterprise's asset risk. Based on the risk orientation of audit fees [18], the main reason for increasing audit fees should be the level of long-term financial asset allocation in financial asset allocation.

Financial assets are characterized by good liquidity, especially short-term financial assets, which enhances the risk aversion ability of enterprises [21]. In order to cope with high costs and financing difficulties brought about by cash flow risks and external financing constraints [22], enterprises tend to hold some short-term financial assets as preventive assets. When the main business development or operation needs funds, and funds are urgently needed to repay the maturing debt, enterprises can quickly liquidate short-term financial assets, reduce the cost of financial distress [12], supplement the required liquidity, enhance the refinancing ability of the enterprise, and effectively reduce the operational risk and financial risk [23]. Enterprise allocation of short-term financial assets is conducive to risk prevention. Therefore, the audited unit has a greater advantage in the audit fee game with the accounting firm, the result of which is that the audit fee will be reduced or remain unchanged.

Unlike short-term financial asset allocations are primarily risk averse, the profit motive may lead firms to allocate to long-term financial assets with higher yields [11, 24]. However, long-term financial assets lack liquidity and the ability to liquidate quickly, when faced with external financing constraints [25], the enterprise's financial risk is relatively high, long-term financial assets invested in a large number of high-risk financial and real estate sectors will increase the enterprise's financial risk, the holding of financial assets has a "risk contagion effect", resulting in higher audit fees [8]. According to the insurance hypothesis, investors regard auditors as the guarantor of investment assets and the payer of investment losses[26, 27], and according to the rational economic man hypothesis, auditors have a strong motivation to avoid risks and make corresponding risk control decisions [28], long-term financial assets have a higher complexity compared to short-term financial assets [29], and their foreign investment is larger in terms of the amount and scope of the investment, and the auditing firms need to put in more human and material resources. The sustained high-quality development of real enterprises can not be separated from the equipment upgrading and reconstruction as well as the research and development of new products and technologies, and if the enterprises excessively hold long-term financial assets with poor liquidity and high conversion costs, the above capital needs can not be effectively met [30, 31]. This can affect the future performance of the entity's main business, leading to financial unsustainability [32, 33], and lack of sufficient collateral for obtaining external credit funds or offsetting maturing debts, which in turn leads to increased business and financial risks [34]. In order to reduce potential future reputational losses, litigation losses and compensation losses [35], auditors have incentives to maintain a more prudent professional attitude and make more robust or conservative decision-making choices for entities holding more long-term financial assets, such as charging higher auditing fees as cost compensation and risk premiums [36]. Higher returns will be faced with higher risks, and profit-seeking motives will cause firms to allocate more of high-risk long-term financial assets.

4. Financial asset allocation has many dimensions, why did the authors only analyze Short-term and Long-term financial asset allocation?

**R: Thank you so much!**

Current research on financial asset allocation has more dimensions, but mostly from the perspective of financialization of enterprises, such as the role of the reservoir and the role of crowding out that affects the normal business operations of enterprises. The main role of financial asset allocation is to obtain more returns, and at the same time, it will bring risks. On the basis of the existing research, in order to further explore the main reasons for the risk of corporate financial asset allocation, this study analyzes from the perspective of long-term financial assets and short-term financial assets, because short-term financial assets, due to the strong liquidity, while long-term financial assets are weakly liquid, the process of obtaining the return will face more uncertainty, so the risk will be more originated from long-term financial assets. This conjecture is also proved in the research of the article.

5. Why the authors chose these moderator variables for Mechanism analysis, further tests are not very relevant to the main logic of the paper's hypothesis, and more tests that are more relevant to the main logic of the paper should be added.

**R: Thank you so much!**

The main responsibility of independent directors is to provide external supervision of enterprises and assist enterprises in making business decisions to help them reduce risks, and the current role of independent directors is getting stronger and stronger. According to the stigma theory, the background characteristics of independent directors will affect the independent directors' judgment and tend to be conservative or aggressive when assisting decision-making, thus affecting the enterprise's business decision-making and enhancing or reducing risks.

In further research we add elements that are more relevant to the main logic of the paper, mainly in terms of firm size and financing constraints to analyze heterogeneity. It is specified as follows:

5.1 Heterogeneity of firm size

Firm size is one of the most basic and important characteristics that affect the investment and financing behavior of firms. It has been found that the trend of financialization of large- size enterprises is more significant [44]. Large- size enterprises have more complex business activities and governance structures, higher agency costs, and are more prone to risks, which in turn affects auditor behavior. Therefore, the sample enterprises are categorized into large-size enterprises and small-size enterprises according to the median enterprise size. The impact of long-term financial asset holdings on corporate audit pricing at different firm sizes is analyzed. The regression results are shown in columns (1) and (2) in Table 8. The results show that the effect of long-term financial assets of firms to increase audit pricing exists in both large-size and small-size firms, but this effect is stronger in large-size firms and passes the test of between-group difference. This implies that larger firms have more complex economic operations and may also face more audit adjustments, resulting in more costs for accountants to obtain more audit evidence and higher audit fees [45].

5.2 Heterogeneity of financing constraints

Firms' financial asset allocations require funding, and in particular, more funding is needed to make long-term financial asset allocations. Therefore, firms will make decisions based on the degree of their financing constraints. If enterprises have more funds and face lower financing constraints, enterprises will increase the possibility of enterprise investing in long-term financial assets; on the contrary, enterprises will reduce the holding of long-term financial assets. Financing constraints have a risk effect, when firms face larger financing constraints, management adopts more aggressive financing strategies, resulting in higher firm risk, therefore, it will have an impact on audit fees. The sample is categorized into high and low financing constraints based on the SA index. Columns (3) and (4) in Table 8 show that there is a more significant positive relationship between long-term financial asset allocation and audit pricing in the higher financing constraint group. The possible reason for this is that when firms face higher financing constraints, they usually adopt aggressive strategies to mitigate the financing difficulties, which raises the risk of the firm. This consequently increases audit costs.

Table 8

| Variables | （1） | （2） | （3） | （4） |
| --- | --- | --- | --- | --- |
|  | LNFEE  Large-Size | LNFEE  Small-Size | LNFEE  High-SA | LNFEE  Low-SA |
| CFin | 0.452^***^ | 0.307^*^ | 0.169^***^ | 0.073 |
|  | (3.27) | (1.76) | (4.43) | (1.49) |
| Cons | 5.302^***^ | 6.096^***^ | 8.035^***^ | 2.309^***^ |
|  | (39.643) | (36.08) | (60.08) | (17.47) |
| Controls | yes | yes | yes | yes |
| Ind/Year | yes | yes | yes | yes |
| *N* | 8547 | 8547 | 8547 | 8547 |
| *R*^2^ | 0.515 | 0.454 | 0.438 | 0.541 |

Note: t-statistics in parentheses, *, **, *** denote statistically significant at 10%, 5%, and 1%.

6. First1 and First2 should not be placed in the control variables at the same time and one of them should be deleted.

**R: Thank you so much!**

Thanks for your suggestion. In the current research on audit fees the control variables mainly contain the percentage of the first largest shareholder or the percentage of the top ten shareholders, this study refers to the selection of control variables in this article “*Du Y, He S, Chen J. Does Financialization Affect Audit Pricing? Auditing Research. 2019;(04):101-10.* ” and cited in our article, First1 and First2 are selected as the control variables in order to increase the reliability of the regression. Thank you very much for your suggestion.

7.Why choose “Investment return/net profit” as instrumental variables? The choice of instrumental variables does not seem to be very convincing.

**R: Thank you so much!**

Thanks for your suggestion. This study refers to the selection of instrumental variables in the following articles and selects “Investment return/net profit” as instrumental variables because since investment return is highly correlated with the level of financial asset allocation and is not intrinsically related to the level of audit input and effort, there is no significant correlation between this instrumental variable and the audit fee obtained from the audit. Because investment return is highly correlated with the level of financial asset allocation and is not intrinsically related to the level of audit effort, this instrumental variable is not significantly correlated with the audit fee obtained from auditing and satisfies the basic conditions of instrumental variables. And references are cited in our article.

*1.Wang H, Cao Y, Yang Q, Yang Z. Does the Financialization of Non-financial Enterprises Promote or Inhibit Corporate Innovation Nankai Business Review. 2017;20(01):155-66. <https://next.cnki.net/middle/abstract?v=Oa1N_PzK0nQAcm79aB4gB7wcTOsF2hGd5E_LQMUAcrKFp5Mv5Du32bgVHABIS4Qd1XEIi7lVw7WnxCRtiEbM79xuxKK0G6hj6p77BY1qhWLe470a_MAmfvqYuFgcJ-NgVvpqUUTsRmaJq6GUnZ-hkW04vgADG4jotWA0xDTS7l1xv5FsoHStdAt8n7vCGnifB3PNNeipvBQ=&uniplatform=NZKPT&language=CHS&scence=null>*

*32.Dong X, Sun W. Enterprise Financialization,Internal Control and Audit Quality. Journal of Audit & Economics. 2021;36(01):26-36. <https://link.cnki.net/urlid/32.1317.F.20210207.1346.024>*

4.3.3. Endogeneity test. Refer to existing research[1, 32], "Investment return/net profit" was selected as IV to test the endogeneity of the research results. Investment return is highly correlated with the level of financial asset allocation, and has no intrinsic correlation with auditor's input and effort, this instrumental variable has no significant correlation with audit fees, meeting the basic conditions of instrumental variables.

8. The endogeneity problem of the article is quite obvious, more endogeneity tests should be added, such as PSM.

**R: Thank you so much!**

Thanks for your suggestion, endogeneity tests and robustness tests were added to the paper, including lagging the dependent variable by one period, performing sample replacement, and also using GMM for endogeneity tests. And the modifications in the paper are as follows:

4.3 Robustness testing

4.3.1 Using alternative variables. Drawing on previous research [32], this study excludes loans and advances issued from long-term financial assets, and the results are shown in Table 4. Columns (1)-(2) show the results of the regression of the term structure of financial asset allocation on audit fees, with the coefficients of SFin1 being insignificant and the coefficients of CFin1 being significantly positive at the 1% level. This suggests that even if the term structure of financial asset allocation is replaced, the findings are still consistent with the main regression.Using lagged independent variables for regression analysis, the results are shown in Table 4. Columns (3)-(4) show the results that the coefficients of SFin2 being insignificant and the coefficients of CFin2 being significantly positive at the 1% level.

4.3.2 Using the fixed effects model. Only year and industry fixed effects are controlled for in the main test model. Here the test is further tested using a two-way fixed effects model, controlling for individual fixed effects. The regression results are shown in Table 4, the coefficient of short-term financial assets in Column (5) is negative but not significant; the coefficient of Column (6) is significantly positive at least at the 5% level, which indicates that the higher the level of long-term financial assets leads to a significant increase in audit fees.

Table 4

| Variables | （1） | （2） | （3） | （4） | （5） | （6） |
| --- | --- | --- | --- | --- | --- | --- |
|  | LNFEE | LNFEE | LNFEE | LNFEE | LNFEE | LNFEE |
| SFin1 | 0.250 |  |  |  |  |  |
|  | (0.84) |  |  |  |  |  |
| CFin1 |  | 0.458^***^ |  |  |  |  |
|  |  | (9.06) |  |  |  |  |
| SFin2 |  |  | 0.203 |  |  |  |
|  |  |  | (1.02) |  |  |  |
| CFin2 |  |  |  | 0.319^***^ |  |  |
|  |  |  |  | (3.47) |  |  |
| SFin |  |  |  |  | -0.109 |  |
|  |  |  |  |  | (-0.49) |  |
| CFin |  |  |  |  |  | 0.165^**^ |
|  |  |  |  |  |  | (2.07) |
| Cons | 4.088^***^ | 4.064^***^ | 3.875^***^ | 3.927^***^ | 4.130^***^ | 4.026^***^ |
|  | (33.63) | (33.61) | (28.36) | (27.54) | (33.62) | (32.08) |
| Controls | yes | yes | yes | yes | yes | yes |
| Ind | yes | yes | yes | yes | no | no |
| Year | yes | yes | yes | yes | yes | yes |
| Company | no | no | no | no | yes | yes |
| N | 8547 | 8547 | 8547 | 8547 | 8547 | 8547 |
| *R*^2^ | 0.687 | 0.690 | 0.514 | 0.506 | 0.679 | 0.680 |

Note: t-statistics in parentheses, *, **, *** denote statistically significant at 10%, 5%, and 1%.

4.3.3. Endogeneity test. Refer to existing research[1, 32], "Investment return/net profit" was selected as IV to test the endogeneity of the research results. Investment return is highly correlated with the level of financial asset allocation, and has no intrinsic correlation with auditor's input and effort, this instrumental variable has no significant correlation with audit fees, meeting the basic conditions of instrumental variables. At the same time, Hansen J statistic P value is 0.167, F value is 549.457, LM statistic is 206.592, indicating that the instrumental variables are valid. Columns (1) and (2) in Table 5 are the test results of the instrumental variable method. The regression coefficient of the first stage IV in column (1) is 0.0219, which is significantly positive at the 1% level, indicating that there is a significant positive correlation between instrumental variables and endogenous variables. In column (2), the regression coefficient of long-term financial assets in the second stage is 0.301, which is significantly positive at the 1% level, indicating that audit pricing increases significantly with the increase of long-term financial assets. The test results are consistent with the above hypothesis.

Meanwhile, the generalized method of moments (GMM) was used to re evaluate the model. Refer to existing research, the lagged one period independent variable was selected as the instrumental variable[40]. The result meet the requirements of overidentification test and autocorrelation test, which is shown in Table 5. In columns (3), the regression coefficient of short-term financial assets is 0.109, there is no significant. Columns (4) shown that the regression coefficient of long-term financial assets is significantly positive at the 5% level, which is confirmed the reliability of the above results.

Table 5

| Variables | （1） | （2） | （3） | （4） |
| --- | --- | --- | --- | --- |
|  | IV-2SLS | | GMM | GMM |
|  | CFin | LNFEE | LNFEE | LNFEE |
| IV | 0.0219^***^ |  |  |  |
|  | (12.78) |  |  |  |
| CFin |  | 0.301^***^ |  |  |
|  |  | (5.13) |  |  |
| SFin |  |  | 0.109 |  |
|  |  |  | (0.96) |  |
| CFin |  |  |  | 0.016^**^ |
|  |  |  |  | (1.99) |
| Cons | 0.0571^**^ | 4.014^***^ | 1.976^**^ | 2.013^***^ |
|  | (2.20) | (27.70) | (2.03) | (16.03) |
| Controls | yes | yes | yes | yes |
| Ind | yes | yes | yes | yes |
| Year | yes | yes | yes | yes |
| Company | no | no | no | no |
| N | 8547 | 8547 | 8547 | 8547 |
| *R*^2^ | 0.246 | 0.680 | 0.427 | 0.549 |

Note: t-statistics in parentheses, *, **, *** denote statistically significant at 10%, 5%, and 1%.

9. 5.2 Financial risks mechanism test should be put into 4.4 Mechanism analysis.

**R: Thank you so much!**

Thanks for your suggestion, we put 5.2 Financial risks mechanism test into 4.4 Mechanism analysis.

10. The literature cited in the paper is not so convincing, and many of them need to be replaced.

**R: Thank you so much!**

Thanks for your suggestion, we have replaced the references with more persuasive ones. and made changes in the paper.

Thank you very much for the specific comments you gave, which not only improved the quality of our manuscript, but also helped us to understand more deeply the research in our field. This is very important for our next research endeavors. Your suggestions are sincerely appreciated!

Thanks again and Best regards!
