# Supplementary material for: Does financial asset allocation term structure affect audit fees? Evidence from China
Source: PLoS One. 2025 Jan 17;20(1):e0317671. doi: 10.1371/journal.pone.0317671 (PMC11741647; doi:10.1371/journal.pone.0317671)
Supplement: Attachment — Submitted filename: Response to Reviewers.docx [file pone.0317671.s003.docx]

**Response to Reviewers**

**Dear Editors and Reviewers:**

Thanks a lot for your letter and the reviewers’ comments concerning our manuscript entitled “*Does financial asset allocation term structure affect audit fees? Evidence from China*”. We are happy to respond to your comments and suggestions, and grateful to take this as a great opportunity to update our information and try our best to meet the high standards of ***PLOS ONE***. The main corrections in the paper and the responses to the editor’s and reviewers’ comments, point-by-point, are as follows:

**Reviewers' comments:**

**R: Thank you so much!**

We believe that your valuable comments are very helpful in improving the quality of the manuscript and have expanded our research vision and research ideas, giving us a deeper knowledge of current research. It helps to improve the level of our manuscript.

**Reviewer #1:**

1. The appropriateness of the term "Audit fees theory" and whether it is clearly defined in the existing literature needs further investigation. It is suggested that the authors provide a more detailed explanation of this theory and review relevant literature.

**R: Thank you so much!**

Thank you for your careful review. The presence of the term audit fee theory in the article is a presentation and translation error. We have revised the expression in the manuscript and adjusted it in the article, and the term "Audit fees theory" was deleted. What we changed in the manuscript is as follows:

Chinese real enterprises face a dilemma characterized by shrinking markets, intensifying competition, overcapacity, profit compression, and declining returns on investment [1, 2]. In the context of risk-oriented auditing, scholars have increasingly explored the relationship between financial asset allocation and audit fees. Audit fees primarily depend on two factors: cost inputs and the risk premium associated with the audit process [3].

2. The significance of conducting the study in the context of China should be further emphasized in the contribution section.

**R: Thank you so much!**

Thanks for your comments, we have changed the Contributions section of the Introduction, and we also have changed the expression of theoretical and practical implications sections of the Conclusions. What we changed in the manuscript is as follows:

Introduction:

This study makes several potential contributions. Firstly, it examines the relationship between financial asset allocation and audit fees from two dimensions: short-term and long-term financial assets, thereby extending the existing research on the relationship between financial asset allocation and audit fees in Chinese firms. Secondly, from the perspective of risk-oriented audit fees, this study finds that within the financial asset allocation term structure, long-term financial asset allocation exposes firms to higher financial risks, which is the primary driver of increased audit fees. This finding provides a rational explanation for the differences in audit fee levels among Chinese listed firms from the perspective of risk-oriented, enriching the literature on the factors influencing audit fee variations. Thirdly, it expands the research on independent director characteristics by examining their role in decision-making and monitoring during firms' financial asset allocation. It also offers empirical insights into preventing the risks associated with financial asset allocation in Chinese firms. This study provides a theoretical foundation for understanding the relationship between the financial asset allocation term structure and audit fees in Chinese firms. Additionally, it offers empirical evidence on strategies corporate managers can adopt to mitigate financial asset allocation risks and reduce audit fees. The findings also have practical implications for accounting firms and government agencies.

Conclusions:

China's economy has become an increasingly significant component of global economic development, with Chinese enterprises playing a vital role in driving economic growth. Against the backdrop of the financialization of Chinese real enterprises, this study holds important theoretical and practical implications. First, it extends the discussion on the economic impact of the financial asset allocation term structure of Chinese firms and the determinants of audit costs. Additionally, it uncovers the intrinsic mechanism linking the financial asset allocation term structure to audit fees, emphasizing the need for firms to focus on optimizing their financial asset allocation—particularly long-term financial assets—to mitigate financial risk and reduce audit fees. Furthermore, the study provides a theoretical foundation for strengthening the role of independent directors in Chinese enterprises. Firms should leverage the oversight and decision-making capabilities of independent directors to mitigate risks associated with financial asset allocation, thereby reducing audit supervision and related costs. Accounting firms are encouraged to prioritize understanding the financial asset allocation term structure to determine audit fees more effectively. Lastly, government authorities should guide real enterprises in optimizing their financial asset allocation structures to foster a virtuous cycle between finance and the real economy.

3. The current endogeneity tests in the paper appears inadequate. The authors are encouraged to add one or two more endogeneity tests.

**R: Thank you so much!**

Thank you for your comment, we have utilized instrumental variable method and GMM for endogeneity test, and the above two methods explain the endogeneity problem of the article.

Instrumental variable method (IV) refers to the literature *Wang H, Cao Y, Yang Q, Yang Z. Does the Financialization of Non-financial Enterprises Promote or Inhibit Corporate Innovation Nankai Business Review. 2017;20(01):155-66.* and *Dong X, Sun W. Enterprise Financialization,Internal Control and Audit Quality. Journal of Audit & Economics. 2021;36(01):26-36. https://link.cnki.net/urlid/32.1317.F.20210207.1346.024*

GMM refers to the literature *Zhou S, Ye N, Zhan W. The impact of corporate financialization on corporate innovation:Moderating role of digital transformation. Statistics & Decision. 2024;40(16):183-8.http://dx.doi.org/10.13546/j.cnki.tjyjc.2024.16.033*

Meanwhile, according to your comment, we added high-dimensional fixed effects and fixed effects considering individual, industry and year in the endogeneity test. To increase the robustness of the findings, we adjusted the sample interval in the robustness test to reduce the effect of the 2008 financial crisis. And the independent variables are lagged one and two periods for the test. And the manuscript was revised. Detailed endogeneity test and robustness test including **Instrumental variable method(IV)**, **GMM**, **High-dimensional Fixed Effect Model**, **Replacing the independent variables, Lagging the independent variables** **by one period and two periods**, **Fixed effect model and Replacing the sample intervals were used in the manuscript**, which took into account the possible scenarios and explained the possible endogeneity and robustness issues, and the results showed that this study is robust. Thank you for your comments, which provide input on the reliability of our study, which we will continue to improve in subsequent studies.

What we changed in the manuscript is as follows:

**4.3 Robustness testing**

4.3.1 Replacement of measures of the independent variable. Drawing on previous research [27], tthis study excludes loans and advances issued from the calculation of long-term financial assets. The results, presented in Table 4, columns (1)–(2) indicate that the coefficients for SFin1 are insignificant, while the coefficients for CFin1 remain significantly positive at the 1% level. These findings are consistent with the main regression results, reinforcing the robustness of the analysis.

4.3.2 Lagged Independent Variable. Lagged the independent variable by one and two periods, the results are shown in Table 4. Columns (3)-(6) show the results that the coefficients of L1.SFin and L2.SFin are insignificant, the coefficients of L1.CFin and L2.CFin are significantly positive at the 1% level and 5% level. These findings confirm the robustness of the main regression results.

4.3.3 Adjusting the sample interval. To mitigate the potential impact of the financial crisis, data from 2009 and 2010 were excluded from the analysis. The results, presented in columns (7)-(8) of Table 4, show that the coefficients for CFin remain significantly positive at the 1% level. These findings further confirm the robustness of the main regression results.

Table 4

|  | （1） | （2） | （3） | （4） | （5） | （6） | （7） | （8） |
| --- | --- | --- | --- | --- | --- | --- | --- | --- |
| Variables | LNFEE | LNFEE | LNFEE | LNFEE | LNFEE | LNFEE | LNFEE | LNFEE |
| SFin1 | 0.250 |  |  |  |  |  |  |  |
|  | (0.84) |  |  |  |  |  |  |  |
| CFin1 |  | 0.458^***^ |  |  |  |  |  |  |
|  |  | (9.06) |  |  |  |  |  |  |
| L1.SFin |  |  | 0.203 |  |  |  |  |  |
|  |  |  | (1.02) |  |  |  |  |  |
| L1.CFin |  |  |  | 0.319^***^ |  |  |  |  |
|  |  |  |  | (3.47) |  |  |  |  |
| L2.SFin |  |  |  |  | 0.143 |  |  |  |
|  |  |  |  |  | (0.28) |  |  |  |
| L2.CFin |  |  |  |  |  | 0.204^**^ |  |  |
|  |  |  |  |  |  | (2.01) |  |  |
| SFin |  |  |  |  |  |  | 0.172 |  |
|  |  |  |  |  |  |  | (0.69) |  |
| CFin |  |  |  |  |  |  |  | 0.134^***^ |
|  |  |  |  |  |  |  |  | (2.99) |
| Cons | 4.088^***^ | 4.064^***^ | 3.875^***^ | 3.927^***^ | 3.106^***^ | 3.437^***^ | 2.517^***^ | 3.313^***^ |
|  | (33.63) | (33.61) | (28.36) | (27.54) | (22.61) | (24.58) | (18.54) | (23.16) |
| Controls | yes | yes | yes | yes | yes | yes | yes | yes |
| Ind/Year | yes | yes | yes | yes | yes | yes | yes | yes |
| N | 8547 | 8547 | 8547 | 8547 | 8547 | 8547 | 7942 | 7942 |
| *R*^2^ | 0.687 | 0.690 | 0.514 | 0.506 | 0.462 | 0.493 | 0.416 | 0.484 |

Note: t-statistics in parentheses, *, **, *** denote statistically significant at 10%, 5%, and 1%.

**4.3.4 Endogeneity test.**

Instrumental Variables (IV) Estimation. Following prior research [1, 27], "investment return/net profit" was selected as the instrumental variable (IV) to address potential endogeneity concerns in the study's findings. Investment return is strongly correlated with the level of financial asset allocation but has no intrinsic relationship with auditor input or effort. This ensures that the IV is uncorrelated with audit fees, satisfying the basic conditions for a valid instrumental variable. At the same time, Hansen J statistic P value is 0.167, F value is 549.457, LM statistic is 206.592, confirm the validity of the instrumental variable. Columns (1) and (2) in Table 5 present the results of the IV method. In the first-stage regression, the coefficient of the IV in column (1) is 0.0219, which is significantly positive at the 1% level, indicating a strong positive relationship between the IV and the endogenous variable. In the second-stage regression, the coefficient of long-term financial assets in column (2) is 0.301, also significantly positive at the 1% level, demonstrating that audit pricing increases substantially with higher levels of long-term financial assets. These results are consistent with the main regression findings.

Generalized Method of Moments(GMM). GMM was used to re-evaluate the results. Following prior research, the lagged one-period independent variable was selected as the instrumental variable [36]. The results satisfy the requirements for overidentification and autocorrelation tests. As shown in Table 5, column (3) reports a regression coefficient of 0.109 for short-term financial assets, which is not statistically significant. Column (4) indicates that the regression coefficient for long-term financial assets is significantly positive at the 5% level, further confirming the reliability of the previous findings

Fixed Effects Model. Endogeneity can arise from omitted variables. To address this, the study incorporates year fixed effects, industry fixed effects, and firm fixed effects in the regression model. The results are presented in Table 5. In Column (5), the coefficient for short-term financial assets is insignificant, while in Column (6), the coefficient is significantly positive at the 5% level. Additionally, a high-dimensional fixed effects regression at the "industry-year" level is conducted, as shown in Columns (7) and (8) of Table 5. The results indicate that higher levels of long-term financial assets significantly increase audit fees, confirming the robustness of the main regression findings.

Table 5

|  | （1） | （2） | （3） | （4） | （5） | （6） | （7） | （8） |
| --- | --- | --- | --- | --- | --- | --- | --- | --- |
|  | IV-2SLS | | GMM | GMM |  |  |  |  |
| Variables | CFin | LNFEE | LNFEE | LNFEE | LNFEE | LNFEE | LNFEE | LNFEE |
| IV | 0.0219^***^ |  |  |  |  |  |  |  |
|  | (12.78) |  |  |  |  |  |  |  |
| CFin |  | 0.301^***^ |  |  |  |  |  |  |
|  |  | (5.13) |  |  |  |  |  |  |
| SFin |  |  | 0.109 |  | 0.312 |  | 0.407 |  |
|  |  |  | (0.96) |  | (1.16) |  | (1.27) |  |
| CFin |  |  |  | 0.016^**^ |  | 0.330^**^ |  | 0.493^**^ |
|  |  |  |  | (1.99) |  | (1.97) |  | (2.41) |
| Cons | 0.0571^**^ | 4.014^***^ | 1.976^**^ | 2.013^***^ | 1.476^***^ | 3.540^***^ | 2.219^***^ | 3.131^***^ |
|  | (2.20) | (27.70) | (2.03) | (16.03) | (23.54) | (26.18) | (18.33) | (19.16) |
| Controls | yes | yes | yes | yes | yes | yes | yes | yes |
| Ind/Year | yes | yes | yes | yes | yes | yes | yes | yes |
| Company | no | no | no | no | yes | yes | no | no |
| Ind -Year | no | no | no | no | no | no | yes | yes |
| N | 8547 | 8547 | 8547 | 8547 | 8547 | 8547 | 8547 | 8547 |
| *R*^2^ | 0.246 | 0.680 | 0.427 | 0.549 | 0.436 | 0.548 | 0.477 | 0.501 |

Note: t-statistics in parentheses, *, **, *** denote statistically significant at 10%, 5%, and 1%.

4. The title and introduction of the paper place significant emphasis on the influence of independent directors. However, in the mechanism analysis section, the characteristics of independent directors are only considered as one aspect of the analysis. It is suggested that the authors either include the analysis of independent directors' characteristics in the hypothesis development section, or adjust the title and introduction to focus specifically on audit fees and financial asset allocation structures. This would improve the clarity and logical coherence of the paper’s structure.

**R: Thank you so much!**

We have restructured the title and introduction of the article, we adjusted the article title to “Does financial asset allocation term structure affect audit fees? Evidence from China”, we condensed the analysis of independent directors characteristics in the introduction, and streamlined the analysis of independent director characteristics in the Mechanisms Analysis section. We improved the clarity and logical coherence of the paper’s structure.

What we changed in the manuscript is as follows:

**Article title:**

“Does financial asset allocation term structure affect audit fees? Evidence from China”

The independent directors characteristics are condensed in the introduction.

In recent years, Chinese government and investors have increasingly emphasized the monitoring and decision-making roles of independent directors. The governance of independent directors influences the relationship between the financial asset allocation term structure and audit fees. However, the direction and magnitude of this influence depend on the characteristics of the independent directors. Consequently, different characteristics of independent directors may have varying impacts on the relationship between the financial asset allocation term structure and corporate audit fees [14].

Meanwhile, We have streamlined the analysis of independent director characteristics in the Mechanisms Analysis section

4.4.2 Independent directors characteristics

Independent directors play a critical role in strategy formulation and addressing procedural uncertainties [37-39]. Effective governance by independent directors enhances corporate decision-making efficiency, improves asset allocation decisions, and optimizes the overall corporate asset structure [40]. The characteristics of independent directors define the structure and distribution of their authority, ensuring professional and independent decision-making and monitoring within the framework of formal governance while safeguarding the interests of all shareholders [37]. Specifically, their governance mechanism enhances both decision-making and oversight capacities. According to higher-order theory, the characteristics of top managers, such as age, gender, educational background, and career experiences, which significantly influence a firm’s strategic choices [41]. This study aims to investigate how the individual characteristics of independent directors moderate the relationship between long-term financial assets and audit fees by improving decision-making efficiency. This study identifies gender, financial experience, overseas experience, and academic background as key proxies for the individual characteristics of independent directors. Using Model (2), it examines the moderating effects of the proportion of women (FID), the proportion of directors with financial backgrounds (FBID), the proportion with overseas experience (OID), and the presence of academic backgrounds (AEX) on the positive association between long-term financial assets and audit fees.

**At the same time**, we reordered the financial risk and independent director characteristics in the mechanism analysis. **4.4.1** for financial risk and **4.4.2** for independent directors characteristics. We improved the clarity and logical coherence of the paper’s structure.

5. In the "Further Research" section, the authors should include tests for coefficient differences between groups.

**R: Thank you so much!**

We conducted tests for coefficient differences between groups. and elaborated in the text and in table 8.

6. The overall quality of the writing needs significant improvement.

**R: Thank you so much!**

We embellished the language of writing and improved the spelling, grammar, clarity, concision and overall readability of this manuscript.

Thank you very much for the specific comments you gave, which not only improved the quality of our manuscript, but also helped us to understand more deeply the research in our field. This is very important for our next research endeavors. Your suggestions are sincerely appreciated!

Thanks again and Best regards!
